# Digital Media Use and Screen Time Exposure Among Youths: A Lifestyle-Based Public Health Concern

**DOI:** 10.7759/cureus.88373

**Published:** 2025-07-20

**Authors:** Muhammad I Khanani, Muhammad R Khan, Mohammed F Farooqi, Jawad Fazal, Zainul Aabideen, Najla S Alkuwaiti

**Affiliations:** 1 Medicine, Burjeel Royal Hospital, Al Ain, ARE; 2 Internal Medicine, Sheikh Tahnoon bin Mohammed Medical City (STMC) and Tawam Hospital, Al Ain, ARE; 3 Faculty of Medicine and Health Sciences, UAE University, Al Ain, ARE; 4 Internal Medicine, Sheikh Tahnoon bin Mohammed Medical City (STMC), Al Ain, ARE; 5 Neurology, Burjeel Medical City, Abu Dhabi, ARE; 6 Oncology, Burjeel Medical City, Abu Dhabi, ARE; 7 Pediatrics, Tawam Hospital, Al Ain, ARE

**Keywords:** adolescents, children, digital media, health outcomes, screen time

## Abstract

The pervasive rise of digital media and screen-based entertainment has transformed the developmental landscape for children and adolescents. The COVID-19 pandemic further amplified screen exposure, exacerbating public health concerns. While digital media offers educational, social, and recreational benefits, growing concerns have emerged regarding its adverse health outcomes. Several international guidelines recommend limited screen time particularly for children under five; however, adherence remains inconsistent. This review combines recent global data and research findings to examine the physical, mental, cognitive, developmental, and emotional health consequences of digital media use and excessive screen time among youth. Physical effects include increased risks of obesity, sleep disturbances, visual impairments, and musculoskeletal pain, particularly with prolonged sedentary behaviors. Mental health outcomes are equally concerning, with excessive screen exposure associated with depression, anxiety, low self-esteem, and, alarmingly, self-harm and suicidal tendencies. Screen time exceeding 2-4 hours per day is consistently linked with increased cognitive and developmental health risks, though the threshold for harm remains debated. Despite some potential benefits of high-quality, interactive content, the evidence highlights the need for balanced media use, age-appropriate limits, and active parental guidance. By integrating findings from international studies and public health recommendations, this review provides a comprehensive foundation for clinicians, educators, and policymakers to develop targeted strategies that promote healthier digital behaviors in children and adolescents.

## Introduction and background

Digital media encompasses any content or communication transmitted through internet-enabled devices such as smartphones, tablets, and computers [1]. On the other hand, screen time refers to the duration of time spent on passive screen-based entertainment such as watching videos or browsing, excluding interactive or physically active screen-based activities [2,3]. Over the past two decades, the widespread adoption and expansion of digital media have deeply integrated screen-based entertainment into the daily lives of children and adolescents. Therefore, children and adolescents today are often referred to as “digital natives,” having grown up in an environment saturated with electronic and digital media [4]. 

Young children are increasingly exposed to mobile gadgets and smart devices from infancy, primarily through video calls to connect with distant family and friends, passive exposure to background television, and use of digital media by caregivers as a tool to calm or manage children’s behavior [5]. A survey in the United Kingdom revealed that nearly one in four (21%) toddlers aged three to four owned their own tablet, while 1% had their own smartphone [6]. Another survey conducted by the National Center for Health Statistics stated that one-half of the teenagers aged 12 to 17 years had more than four hours of daily screen time between July 2021 and December 2023 [7]. The COVID-19 pandemic further intensified concerns over screen time, with studies reporting a 52% increase in screen exposure among youth during lockdowns [8]. A systematic review of 53 studies was conducted by Qi et al. (2023) to assess the screen time among school-aged children aged 6 to 14 years and found an estimated average screen time of 2.77 hours per day, with 46.4% exceeding two hours daily. Notably, screen time ≥2 hours/day rose from 41.3% before to 59.4% after the COVID-19 outbreak [9]. The prevalence of excessive screen time among children shows wide variation globally, ranging from 10% to 93.7% in high-income countries and from 21% to 98% in middle-income countries [2,10].

Childhood to adolescence represents a critical developmental window physically, cognitively, socially, and emotionally. Behaviors established during this stage may have long-lasting implications on their health and well-being. This correlation has spurred significant public health interest in whether digital media is a contributing risk factor to various physical and mental health issues in youth. Positive aspects include communication through messaging, exposure to uplifting content for learning, information dissemination, and support from online communities [11]. However, a growing body of evidence links excessive screen time to negative outcomes. For instance, meta-analyses and longitudinal studies suggest that excess screen time leads to obesity [12], cardiometabolic risks, sleep disturbances [13], musculoskeletal complaints [14], depressive symptoms [15], body image concerns, anxiety [16], low self-esteem [17], and poor academic performance [3]. Additionally, daily screen time exceeding 2-4 hours has been associated with long-term health effects. Some studies suggest that negative effects may begin after just 30 minutes of daily use, while others report no adverse outcomes until beyond 3.5 hours [18]. However, there is no consensus on the threshold at which screen time becomes harmful. Interestingly, despite the evidence, many parents believe that screen time promotes creativity and imagination, although most households still report having screen time rules in place [19,20]. 

Based on the complex, contradictory findings, the health consequences of screen exposure in children and adolescents remain inadequately understood. This review aims to synthesize the current evidence regarding the physical, mental, and behavioral health effects of screen time and digital media use in children and adolescents, providing a foundation for informed public health strategies and parental guidance.

## Review

International guidelines on digital media use and screen time

Table 1 provides an overview of recent international recommendations on digital media use and screen time. These guidelines consistently highlight standards related to time restrictions and lifestyle adjustments, emphasizing the importance of setting age-appropriate boundaries, encouraging a healthy balance between screen use and other beneficial activities, and promoting positive family interactions [21]. 

**Table 1:** International guidelines and recommendations on digital media and screen time usage for children and adolescents.

International Organization	Age Group	Screen Time	Additional Recommendations
World Health Organization (WHO) [22]	0–1 year	No screen time	Only allow video chatting with supervision
	1–2 years	No screen time for 1-year-olds; <1 hour/day for 2-year-olds	Less is better; focus on interactive activities
	3–4 years	<1 hour/day	Encourage physical activity and adequate sleep
American Academy of Pediatrics (AAP) [23,24]	<18 months	Avoid screen time	Video chatting is acceptable with adult support
	18–24 months	If introduced, high-quality content only	Watch together and help children understand what they see
	2–5 years	≤1 hour/day of high-quality content	Co-view and discuss content
	≥6 years	Consistent limits; no specific time	Avoid displacement of sleep, physical activity, and family time
Indian Academy of Pediatrics (IAP) [25]	<2 years	Avoid screen exposure	
	2–5 years	≤1 hour/day	Educative interactive content under parental supervision
Canadian Paediatric Society (CPS) [26]	<2 years	Avoid screen time (except video calls)	Focus on interactive, non-screen activities
	2–5 years	<1 hour/day	Encourage co-viewing and active engagement
	≥5 years	Consistent limits; no specific max	Ensure screen use does not interfere with sleep, exercise, learning
National Association for the Education of Young Children (NAEYC) [27]	0-2 years	Avoid screen exposure.	Prohibit passive screen use
	2–5 years	≤1 hour/day	Encourage technology use that supports creative, hands-on, social learning
Australian Department of Health [28]	0–2 years	No sedentary screen time (except video chat)	Promote active play and caregiver interaction
	2–5 years	≤1 hour/day	Break up long periods of sitting
	5–17 years	≤2 hours/day (recreational)	Exclude educational use; encourage outdoor activity

World Health Organization (WHO)

In 2019, WHO published “Guidelines on Physical Activity, Sedentary Behaviour and Sleep for Children under Five Years of Age” recommending age-specific screen time limits for young children [22]. The WHO strongly advises against screen exposure for infants under one year. Similarly, children under two years of age should have no exposure to screen-based media. For children aged two to four years, screen time should be limited to no more than one hour per day. These guidelines also align with those set by the Commission on Ending Childhood Obesity [29] and the Global Action Plan on Physical Activity 2018-2030 [30].

American Academy of Pediatrics (AAP)

In 2016, the AAP released two policy statements entitled “Media Use in School-Aged Children and Adolescents” [23] and "Media and Young Minds" [24] that focus on screen time usage in children and adolescents aged 5 to 18 years. Screen use is discouraged entirely for children under 18 months, except for video chatting to maintain connections with distant family members. For children aged 18-24 months, educational apps may be cautiously used under adult supervision to promote interactive learning. The policy advises that children between two and five years old should have no more than one hour of screen time daily, ideally involving ‘high-quality and age-appropriate programming’ that parents co-watch together with their children. Additionally, parents are advised against relying solely on screen media to calm or distract children and should avoid pressuring early screen use. Parents are encouraged to monitor the quality and content of media their children engage with and to create screen-free environments during key daily routines such as mealtimes, playtime, and at least one hour before bedtime [23,24,31]. 

Indian Academy of Pediatrics (IAP)

The IAP recommends avoiding screen exposure for children under two years old. For children aged two to five years, screen time should be limited to a maximum of one hour per day, with emphasis on educational interactive programs under parental supervision. The guidelines also highlight the importance of ensuring screen time does not replace adequate physical activity, sleep, and interactive social play, and strongly recommend screen-free meals and no screen use at least one hour before bedtime to support healthy development [25].

Canadian Paediatric Society (CPS)

The 2019 and 2022 position statements entitled, “Digital media: Promoting healthy screen use in school-aged children and adolescents” [32] and “Screen time and preschool children: Promoting health and development in a digital world” [26] respectively provide guidance on promoting healthy screen use in school-aged children (5 to 12 years old) and adolescents (up to 19 years). The CPS recommends that children under two years of age should not be exposed to screen time except for video chatting with caring adults. For children aged two to five years, sedentary screen time should be limited to one hour or less per day and should not become a routine part of child care environments. Families are encouraged to establish screen-free times daily during meals and shared reading, and to avoid screen use for at least an hour before bedtime for better sleep. To reduce potential risks, caregivers should be present and actively engaged during screen use, co-viewing content to promote digital literacy and encourage children to critically evaluate advertisements and stereotypes. Prioritizing educational, age-appropriate, and interactive content is essential [26,32].

National Association for the Education of Young Children (NAEYC)

In the 2012 position statement entitled, "Technology and Interactive Media as Tools in Early Childhood Programs Serving Children from Birth through Age 8", the NAEYC emphasized that technology and interactive media can support effective learning and development only when applied within developmentally appropriate frameworks. The focus is on the quality of content, the experience of the child, and the opportunity for co-engagement with caregivers or educators. The NAEYC discourages passive use of non-interactive media for children aged 2 to 5 years. Instead, technology should be used to extend children’s active, creative, hands-on learning and promote engagement with the real world. For children under 2, any screen-based interactions must strengthen adult-child relationships and support responsive communication [27].

Australian Department of Health

The Australian 24-Hour Movement Guidelines, developed by the Australian Department of Health, provide clear age-based screen time limits to promote healthy growth and development in children. For children under two years of age, screen time is not recommended at all, except for activities such as video chatting with family. For those aged 2 to 5 years, recreational screen time should be limited to no more than one hour per day. As children grow older, the recommendation adjusts accordingly: for children aged 5 to 17 years, recreational screen time should not exceed two hours per day [28,33]. 

Despite these official guidelines discouraging early exposure, many children encounter screen time during infancy and develop consistent and ingrained screen use habits by the time they reach preschool age [34]. 

Impact of excessive screen media on child and adolescent health

**Figure 1:**
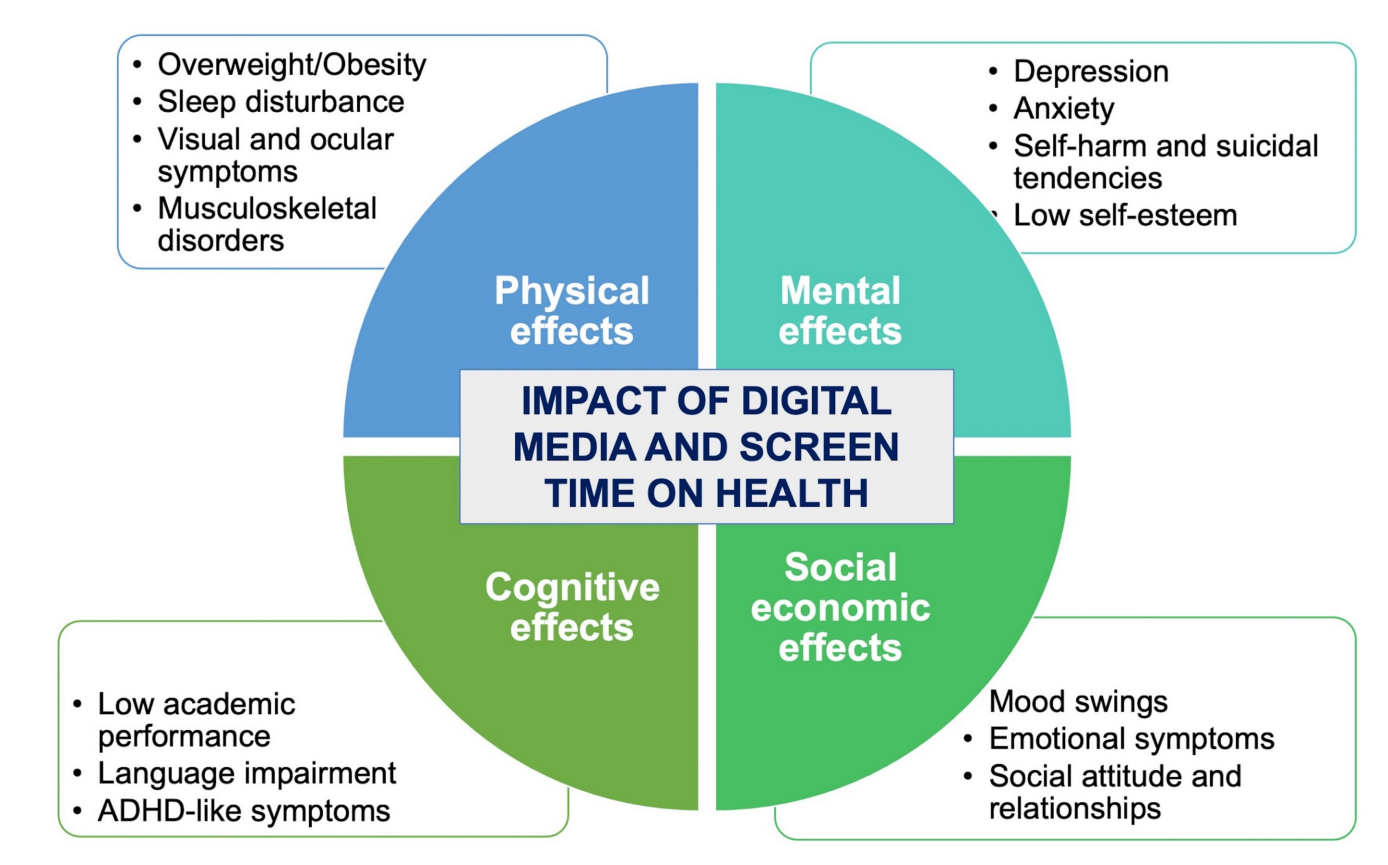
Impact of digital media and excessive screen time on the health of children and adolescents. ADHD: Attention-Deficit/Hyperactivity Disorder Source: Created by the author

Increased media consumption, excessive use of digital devices, and reduced outdoor activities contribute to negative effects on the overall health and development of children and adolescents [5]. Digital technology plays a major role in their lives, influencing their cognitive, mental, physical, and socio-emotional growth in the following ways (Figure 1).

Physical Health

Physical activity is consistently linked with better health outcomes for children and adolescents, while excessive sedentary behavior, including screen time, is associated with less favorable health outcomes [35]. Screen media can promote physical activity through age-appropriate interactive content like dance, yoga, or nature-exploration apps. Active video games and movement-based programs designed for young children can also encourage light to vigorous physical activity [36]. High-quality content can also connect on- and off-screen experiences, supporting imaginative play and social interaction. However, concerns still persist that early screen exposure can lead to sedentary habits and increased risk of overweight/obesity, sleep disruption, and vision impairment. Table 2 summarizes key studies on physical health outcomes associated with screen time in children and adolescents.

**Table 2:** Key studies on physical health outcomes associated with screen time in children and adolescents.

Study (Author, Year)	Population/Sample	Study Design	Key Findings
Obesity			
Nagata et al. (2023) [37]	5,797 U.S. adolescents (10–14 y)	Cross-sectional (ABCD)	Medium (>4–8 hrs) and high (>8 hrs) screen time ↑ risk of overweight/obesity vs. low screen time.
Guzmán et al. (2022) [13]	4,285 European children	Prospective cohort	Each additional hour of screen time and less sleep linked to a higher incidence of overweight/obesity.
Haghjoo et al. (2022) [38]	Meta-analysis of 44 studies	Meta-analysis	Screen time positively associated with obesity risk; 1.27 times increased risk.
de Lima et al. (2020) [39]	Adolescents (sample size not given)	Cross-sectional	Non-significant reduced risk of overweight with >4 hrs/day screen time (P=0.87).
Lopez-Gonzalez et al. (2020) [40]	Mexican children/adolescents	Population-based	No significant direct association of screen time with obesity; combined inactivity and poor sleep ↑ risk.
Arrona-Cardoza et al. (2022) [41]	(Review / observational)	Review	Commercial TV ads promote unhealthy eating, increasing obesity risk.
Sleep Disturbances			
Zhong et al. (2025) [42]	>122,000 adults	Cross-sectional	Screen use before bedtime linked to later bedtimes, ~50 min less sleep/week, 33% higher poor sleep quality.
Nagata et al. (2023) [37]	10,280 adolescents (10–14 y)	Cross-sectional (ABCD)	Bedtime screen use associated with trouble falling/staying asleep and overall sleep disturbances.
Pham et al. (2021) [43]	369 Vietnamese university students	Cross-sectional	48.8% had poor sleep quality; 98.1% used devices within 2 hrs before bedtime.
Janssen et al. (2020) [44]	Infants, toddlers, and preschoolers	Meta-analysis	Screen time associated with poor sleep in infants and toddlers but not in preschoolers.
Visual and Ocular Symptoms			
Alah et al. (2025) [45]	Students in Qatar	Cross-sectional	School closures ↑ screen time by 11.5 hrs; visual acuity declined by 1.7 times.
Kim et al. (2025) [46]	2,064 children (2–17 y)	Retrospective observational	Marked acceleration in myopia progression during the COVID-19 pandemic.
Zong et al. (2024) [47]	Meta-analysis of 19 studies	Meta-analysis	Higher screen time linked to increased myopia risk (OR 2.24).
Zhong et al. (2025) [48]	4,649 children in Shenzhen, China	Population-based	Adherence to movement guidelines including limiting screen time ↓ risk of myopia (aOR=0.73).
Champagne-Hamel et al. (2023) [49]	Mother-child cohort, France	Prospective cohort	Moderate screen exposure at 6 y associated with better contrast sensitivity (girls) and color vision (boys) at 12 y.
Musculoskeletal Disorders			
Lazić et al. (2021) [14]	Adolescents (ages not specified)	Review / cross-sectional	Electronic device use linked to musculoskeletal pain.
Queiroz et al. (2018) [50]	Adolescents	Cross-sectional	Similar associations with musculoskeletal pain.
Nochian et al. (2024) [51]	Medical students	Cross-sectional	Excessive Internet use linked to higher musculoskeletal pain in the neck, wrist, back, hips, and thighs.
Tsang et al. (2023) [52]	1,000+ students, Hong Kong	Cross-sectional	Excessive device use and gaming addiction linked to higher pain rates and severity.
Cankurtaran et al. (2022) [53]	1,000+ students, Turkey	Cross-sectional	Similar findings of higher pain and poorer psychosocial health linked to device use.

Obesity: Sedentary lifestyle in children is often associated with higher body mass index (BMI), body fat, and overweight/obesity [3,54]. In a cross-sectional analysis from the Adolescent Brain Cognitive Development (ABCD) Study involving 5,797 U.S. adolescents aged 10 to 14, Nagata et al. (2023) found that both high screen time and low physical activity were independently associated with increased risk of overweight and obesity. In addition, adolescents reporting medium (>4-8 hours/day) or high (>8 hours/day) screen time had significantly higher risk ratios for overweight or obesity compared to those with low screen time (0-4 hours/day) [55]. In a prospective study of the IDEFICS/I.Family cohort of 4,285 European children, Guzman et al. (2022) examined the association of screen time and sleep duration independently with the incidence of overweight and obesity. Among children who were of normal weight at baseline, each additional hour of screen time and each hour less of sleep were linked to a significantly higher risk of becoming overweight or obese (OR = 1.16 and OR = 1.23, respectively) thereby, increasing the incidence by 13-20% [13]. In a meta-analysis of 44 eligible studies, Haghjoo et al. (2022) evaluated and found a positive association between screen time and obesity risk among adolescents by increasing a risk by 1.27 times. However, the authors did not find any dose-response evidence between screen time and obesity risk [38]. Contrary to these studies, de Lima et al. (2020) evaluated the association between overweight risk and screentime in adolescents and found a non-significant reduced risk of excess weight and increased screen time of more than four hours per day (P = 0.87; 95% CI = 0.59-1.30) [39]. Another population-based study by Lopez-Gonzalez et al. (2020) found no significant association between obesity and screen time among Mexican children/adolescents in a school setting; however, the combination of physical inactivity, increased screen time, and insufficient sleep had the highest risk association with obesity and overweight [40]. Commercial television ads promote unhealthy eating habits through advertising, increasing the risk of poor diet and excessive food intake, which are linked with obesity and overweight [41,56]. 

Sleep disturbances: Adequate sleep plays an imperative role in the overall health and development of children, especially in the early years. In a cross-sectional study of over 122,000 adults from the American Cancer Society Cancer Prevention Study-3, Zhong et al. (2025) found that daily screen use before bedtime was linked to later bedtimes, shorter sleep duration (about 50 minutes less per week), and a 33% higher prevalence of poor sleep quality [42]. Another national study of 10,280 adolescents (aged 10-14) from the ABCD study found that bedtime screen use including having devices in the bedroom, leaving phone ringer on overnight, and engaging in activities like streaming, gaming, or texting, was significantly associated with trouble falling/staying asleep and overall sleep disturbances [37]. A study by Pham et al. (2021) on 369 Vietnamese university students found that 48.8% experienced poor sleep quality, with 98.1% using electronic devices within two hours before bedtime [43]. In a meta-analysis of 31 studies, Janssen et al. (2020) found that screen time is generally associated with poorer sleep outcomes in infants (0-1 y) and toddlers (1-2 y); however, no such association was observed in preschoolers (3-4 y) [44]. The underlying theories for the association of screen use before bedtime and sleep disruptions are due to melatonin suppression, mental arousal, and less playing or physical activities among children and adolescents [37,43].

Visual and ocular symptoms: With prolonged screen exposure due to remote learning and limited outdoor activity, the prevalence of vision-related impairments has increased among children and adolescents. It includes dry eyes, itching, blurred vision, myopia, and headaches [57]. Effective management includes limiting daily screen time, frequent blinking, maintaining proper lighting, and adopting the 20-20-20 rule (looking at something 20 feet away for 20 seconds every 20 minutes). A study by Alah et al. (2025) determined the impact of screen time on the visual acuity of students during school closures in the pandemic in Qatar. The authors found that school closures significantly increased the screen time of the students by 11.5±11.6 hours and were associated with a notable decline in visual acuity by 1.7 times [45]. Another retrospective observational study conducted in 2064 patients aged 2-17 years by Kim et al. (2025), the authors observed a marked acceleration in myopia progression (F-ratio=14.4, p<0.0001) among children during the COVID-19 pandemic due to increased screen time for remote learning and reduced outdoor activity due to home confinement [46]. In a systematic review and meta-analysis of 19 studies, Zong et al. (2024) examined the association between screen time exposure and myopia among children and adolescents. The authors found a statistically significant link between higher screen time and increased risk of myopia (OR: 2.24; 95% CI: 1.47-3.42), indicating a dose-response relationship [47]. Report in the literature suggests that adherence to the Canadian 24-hour movement guidelines, especially limiting screen time and engaging in physical activity, was significantly associated with reduced risks of myopia (aOR = 0.73, 95% CI = 0.56-0.97) and myopic anisometropia (aOR = 0.60, 95% CI = 0.41-0.89) among 4,649 children and adolescents in Shenzhen, China [48]. In contrast, a study by Champagne-Hamel et al. (2023) on the mother-child PELAGIE cohort in France found that moderate screen exposure at age six was associated with better contrast sensitivity at age 12 in girls and improved tritan-axis color vision in boys. These results suggest that moderate screen use in middle childhood may not harm, and could potentially benefit, certain aspects of visual function development [49]. 

Musculoskeletal disorders: Idiopathic musculoskeletal pain affects 30-65% of adolescents and is a leading cause of non-inflammatory pain in this age group. While often underdiagnosed, these pain syndromes can impact daily functioning. Electronic device use has been identified as a potential risk factor [14,50]. A cross-sectional study implicated that excessive Internet use among medical students is linked to a higher risk of musculoskeletal pain in areas such as the neck, wrist, upper back, hips, and thighs due to improper posture and excessive screen time [51]. In two independent large cross-sectional studies of over 1,000 students in Hong Kong (ages 9-17 years) and Turkey, Tsang et al. (2023) and Cankurtaran et al. (2022) found that excessive electronic device use and digital game addiction were significantly associated with higher rates and severity of musculoskeletal pain (neck, wrist, back, and lower back), visual symptoms, and poorer psychosocial health, respectively. Adolescents reported more severe effects than younger children [52,53]. Another cross-sectional study of 299 adolescents found that musculoskeletal pain was more common among older students, female subjects, and those using multiple electronic devices, particularly cell phones [50].

Mental Health

Over the past two decades, a growing body of research has examined the influence of digital media use on the mental development of children and adolescents [5,58,59]. Across the diverse settings, the emerging pattern suggests that excessive recreational screen time is consistently associated with poorer mental health outcomes in adolescents, including depression, anxiety, low self-esteem, life satisfaction, self-harm, and suicidal tendencies [60]. Table 3 summarizes key studies examining associations between screen time and adolescent mental health outcomes.

**Table 3:** Key studies summarizing associations between screen time and adolescent mental health outcomes.

Study	Country	Sample Size / Design	Screen Time Exposure	Mental Health Outcomes	Key Findings
Frielingsdorf et al., 2025 [17]	Sweden	3,566 adolescents	>4–6 hours/day digital media (entertainment)	Depression, anxiety, self-esteem, pain, sleep	Dose-response relationship with adverse outcomes
Gao & Gao, 2024 [61]	Meta-analysis (9 studies)	Prospective cohort	General screen time	Depression	OR = 1.20 (95% CI: 1.12–1.28); +1 hour/day increases risk
Chen et al., 2022 [16]	China	1,331 adolescents (48.7% girls)	≥6 hours/day video games	Anxiety (by gender)	Significant association for boys (OR=5.12), not girls
Kidokoro et al., 2022 [62]	Japan	—	≥5 hours/day screen time	Anxiety, depression	Significant effects in girls, not boys
Kjellenberg et al., 2022 [63]	Sweden	—	≥5 hours/day screen time	Anxiety, depression	Significant in girls only
Marciano et al., 2022 [64]	Switzerland	Longitudinal	Social media vs. TV	Anxiety, depression, inattention	Social media ↑ risk; TV ↓ anxiety/inattention
Gilchrist et al., 2021 [65]	Canada	—	Reallocation of 15 min from ST	Depression, anxiety	Substitution with PA/sleep/homework improves outcomes
Chu et al., 2023 [66]	USA (ABCD Study)	11,633 children (9–11 yrs)	+1 hour/day screen time	Suicidal behaviors (2 years later)	+9% odds per extra hour
Rasmussen et al., 2025 [67]	Denmark	28,613 adolescents	≥6 hours/day screen time	Suicidal ideation, attempts	aRRR: 1.67 in females (ideation); attempts in both sexes
McAllister et al., 2021 [68]	UK	—	>3 hours/day social media	Self-harm, depression	Significant in girls; not boys or other media types
Twenge & Campbell, 2018 [69]	USA	>200,000 adolescents	>3 hours/day device use	Suicide-related outcomes	34% higher risk than ≤1 hour/day; dose-response observed
Orben et al., 2022 [70]	UK	17,409 adolescents	Digital tech use	Life satisfaction, self-image	Negative association, esp. in girls
Sampasa-Kanyinga et al., 2022 [71]	Canada	>28,000 adolescents	≥2 hours/day recreational ST	Poor mental health, low satisfaction	Strong negative associations
Boer et al., 2020 [72]	29 countries	Cross-national sample	Social media use	Body/life dissatisfaction, anxiety	Girls more negatively affected
Suchert et al., 2015 [73]	Germany	—	Screen-based sedentary behavior	Depression, self-esteem	Girls: ↓ self-esteem; Boys: ↑ self-esteem

Depression and anxiety: Multiple studies across diverse countries have examined the relationship between different types of screen-based sedentary behavior and symptoms of anxiety and depression in adolescents [15,59]. For instance, a Swedish study of 3,566 adolescents found that spending over 4-6 hours daily on digital media (especially for entertainment) was linked to poor self-esteem, depression, anxiety, pain, and sleep problems, suggesting a dose-response relationship between screen time and adverse health outcomes [17]. In a meta-analysis of nine prospective cohort studies, it was found that increased screen time is significantly associated with a higher risk of depression in adolescents (OR = 1.20, 95% CI: 1.12-1.28). Further, an hour per day increase in screen time further intensified the depression risk [61]. Several studies have highlighted gender-specific vulnerabilities to digital media exposure [16,62,63]. In China, Chen et al. (2022) conducted a cross-sectional study with 1,331 adolescents (48.7% girls) and found that spending six or more hours daily on video games was strongly associated with increased anxiety symptoms (OR=5.12) among boys, whereas no significant anxiety associations were observed in girls [16]. Conversely, Kjellenberg et al. (2022) and Kidokoro et al. (2022) in Sweden and Japan independently reported that high overall screen time (≥5 hours/day) was significantly linked to increased anxiety and depressive symptoms in girls but not in boys [62,63]. Notably, television viewing was associated with lower depression rates, indicating that not all screen time impacts mental health equally [59,61]. In Switzerland, Marciano et al. (2022) longitudinally observed that increases in social media use predicted poorer mental health including anxiety and depression over time, while time spent watching television was related to reduced anxiety and inattention symptoms, further supporting activity-specific effects [64]. Large-scale Canadian data analyzed by Gilchrist et al. (2021) demonstrated that reallocating even 15 minutes from screen time to physical activity, sleep, or homework modestly improved mental health outcomes including depression and anxiety [65].

Self-harm and suicidal tendencies: Suicide is one of the leading causes of mortality among adolescents. Emerging literature suggest a significant association between screen time, social media usage, and suicidal behaviors [59,66,74]. In a national cohort study among 28,613 Danish adolescents, Rasmussen et al. (2025) found daily screen time of ≥ 6hours was associated with suicidal ideation in females (aRRR:1.67, 95% CI:1.44-1.93) and suicide attempt in both sexes [67]. Another prospective ABCD cohort study of 9-11 year olds (N = 11,633) in the US demonstrated that each additional hour of daily screen time was associated with a 9% increase in the odds of suicidal behaviors two years later [66]. In the UK, McAllister et al. (2021) reported that girls spending over three hours daily on social media had significantly higher rates of self-harm and depressive symptoms compared to those with less than one hour, whereas no significant associations emerged for boys or other screen activities [68]. Another large-scale review by Twenge and Campbell (2018), which analyzed data from over 200,000 adolescents in the United States, reported that adolescents who spent more than three hours daily on electronic devices were 34% more likely to suffer from at least one suicide-related outcome compared to those with one hour or less of screen time. Their findings support a dose-response relationship in which the risk of poor mental health increases with each additional hour of screen use [69].

Self-esteem and self-image: A UK-based study by Orben et al. (2022) applied advanced modeling techniques to data from the Millennium Cohort Study (n=17,409) and found that increased digital technology use was significantly associated with a decrease in life satisfaction ratings and self-image among adolescent girls [70]. In Canada, Sampasa-Kanyinga et al. (2022) conducted a cross-sectional study involving over 28,000 adolescents and found that those engaging in ≥2 hours/day of recreational screen time were significantly more likely to report poor mental health, low life satisfaction, and low self-rated mental well-being [71]. Boer et al. (2020), using a cross-national sample of 29 countries, found that girls were more negatively affected by social media use in relation to life and body dissatisfaction, and social anxiety [72]. In Germany, Suchert et al. (2015) observed that screen-based sedentary behaviors predicted higher depression and lower self-esteem among girls, while boys paradoxically showed higher self-esteem with increased screen use [73].

Cognitive and Developmental Effects

Screen media can influence the cognitive development of children and adolescents in both positive and negative ways. On the beneficial side, educational media tools such as e-books and reading apps may enhance early literacy skills and promote creative thinking [75]. However, several studies have also documented negative impacts on cognitive domains including executive functioning, sensorimotor skills, and academic performance [4,76]. Media multitasking, in particular, has been linked to reduced working memory, poor inhibitory control, and difficulty switching between tasks [77]. Table 4 summarizes key studies related to screen time effects on cognitive and developmental outcomes in children and adolescents.

**Table 4:** Key studies summarizing screen time effects on cognitive and developmental outcomes in children and adolescents. ADHD: Attention-Deficit/Hyperactivity Disorder, TV: Television, hr: hour, AOR: Adjusted Odds Ratio

Study	Country	Design / Sample	Screen Time Exposure	Key Findings
Academic Performance				
Salari et al., 2025 [78]	-	Systematic review & meta-analysis	Social media addiction	Significant negative correlation (r = -0.172)
Adelantado-Renau et al., 2019 [79]	-	Meta-analysis (N = 480,479)	TV & video games	TV/video games linked to poorer academic scores
Mao et al., 2022 [80]	China	Cross-sectional (n = 1,385 college students)	>60 min post-10 PM screen use	Higher risk of poor academic performance (AOR = 1.28)
Muppalla et al., 2023 [4]	U.S.	Observational study	Media multitasking	Negative associations with academic outcomes
Peiró-Velert et al., 2014 [81]	Spain	Observational study	Screen time and multitasking	Poorer performance with higher screen use
Pagani et al., 2010 [82]	Canada	Longitudinal (Quebec Study)	TV at age 2	1hr/day ↑ TV → lower math and participation at Grade 4
Language Impairment				
Bhutani et al., 2024 [83]	Global (16 studies)	Scoping review	General screen time	9/16 studies found negative effects; 2 positive
Al Hosani et al., 2023 [84]	UAE	Case-control (children aged 12–48 months)	Early and frequent use of smartphones/tablets	90.3% of delayed cases had high device exposure
Martinot et al., 2021 [85]	France	Longitudinal cohort study	>2–3 hours/day screen use	Linked to weaker vocab & more behavior issues
ADHD-like Symptoms				
Abdoli et al., 2025 [86]	-	Systematic review (8 studies)	Problematic digital media use	Bidirectional link between media use and ADHD
Thorell et al., 2024 [87]	-	Systematic review (28 longitudinal studies)	Digital media use	Digital media affects sleep/social factors contributing to ADHD
Wallace et al., 2023 [88]	Canada	5-year longitudinal (n ≈ 4,000)	Screen/social media use	ST increase = ↑ impulsivity, ↓ working memory
Shuai et al., 2021 [89]	China	Observational study	Video games/social media	Media use linked to worse ADHD, stress, and family cohesion

Academic performance: A systematic review and meta-analysis conducted by Salari et al. (2025) found a significant negative impact of social media addiction on academic performance of the university students (r=-0.172, 95% CI: -0.320 to -0.016), highlighting that excessive use of social networks can detract from students’ academic outcomes [78]. Another systematic review and meta-analysis of 58 studies with 4,80,479 participants found that while overall screen time was not significantly associated with academic performance, specific activities like television viewing and video game playing were linked to lower scores in language, mathematics, and composite academic outcomes. These associations were more pronounced in children for language and adolescents for composite scores [79]. The cross-sectional study among 1,385 Chinese college students conducted by Mao et al. (2022) found that using electronic screens for entertainment for more than 60 minutes after 10:00 p.m. (pre-bedtime) was significantly associated with poor academic performance (AOR = 1.28) and poor sleep quality (AOR = 1.87) [90]. Studies from Spain and the U.S. found that increased screen time and media multitasking were associated with poorer academic outcomes, particularly in mathematics and English [4,81]. Longitudinal data from the Quebec Longitudinal Study of Child Development showed that each additional hour of television watched daily at age two predicted reduced classroom participation and lower math proficiency in fourth grade [82].

Language impairment: Early childhood is a critical period for language acquisition, which relies heavily on interactive communication with adults [91]. Excessive screen time has been associated with reduced parent-child interactions, thereby limiting opportunities for children to practice language skills [92]. In a scoping review, Bhutani et al. (2024) examined the relationship between screen time and language development in children under 12 years of age. Out of 16 included studies, nine studies found a negative impact of screen time on language development, while five found no significant effect, and two reported positive outcomes. The review suggests that excessive unsupervised and non-educational media screen time may hinder language acquisition in young children [83]. A case-control study demonstrated that early and frequent use of electronic devices (smartphones, tablets) in children aged 12-24 months is significantly associated with speech and language delays in preschool children (90.3% cases). In contrast, traditional TV viewing and higher maternal education were less strongly linked to such delays [93]. While excessive screen time in early years is linked to delays in language and social development, starting screen use later in childhood and engaging in co-viewing with caregivers can yield some benefits [94]. Co-viewing has been associated with improved expressive vocabulary, phonological skills, and overall language development. Conversely, children exposed to more than 2-3 hours of screen time daily tend to show more behavioral problems and weaker vocabulary acquisition [95]. Despite concerns, digital platforms such as blogs and educational websites are also being effectively used in schools to support language and literacy development [96].

Attention-deficit/hyperactivity disorder (ADHD)-like symptoms: Accumulating evidence in the literature suggests that digital media use and screen time are associated with ADHD symptoms [76,87,97]. For instance, two independent systematic reviews of eight articles and 28 longitudinal studies, respectively found evidence of reciprocal associations between problematic digital media use and ADHD symptoms in children and adolescents by impacting sleep cycles and social relationships [86,87]. In a five-year longitudinal study of nearly 4,000 Canadian high school students, Wallace et al. (2023) found that increases in screen time, especially social media use, are associated with worsening ADHD symptoms such as impulsivity, response inhibition on a Go/No-Go task, and working memory, in adolescents within the same year [88]. Another study by Shuai et al. (2021) found that those children with problematic digital media use spent more time on video games and social media and showed more severe ADHD symptoms, greater emotional problems (anxiety, depression), executive function deficits, higher stress, lower learning motivation, and worse family cohesion during the COVID-19 pandemic [89].

Social-Emotional Effects

Screen exposure can affect social-emotional growth. For example, a large-scale study involving 6,623 preschool children in China found that excessive digital screen exposure was significantly linked to more severe emotional symptoms (β=0.2351, p<0.01) and this association was mediated by the parent-child relationship [98]. In a longitudinal study, Rodman et al. (2024) tracked adolescents' smartphone use and mood fluctuations over 12 months using digital phenotyping and found that improved mood led to increased use of communication apps, while greater entertainment app use predicted better mood. However, increased screen time or social media use was not linked to worse mood, emphasizing the relationship between smartphone usage pattern and emotional impacts [99]. A qualitative study explored that active engagement promoted social connectivity and positive emotions, while passive use was linked to more stress. The impact of screen use depended on the type and purpose of engagement, highlighting the importance of understanding how adolescents use digital media [100]. Furthermore, excessive screen time at age four has been associated with reduced emotional understanding by age six and having a television in the bedroom at age six has been linked to lower emotional understanding by age eight. Gender differences have also been observed where boys who engage in gaming appear more likely to experience reduced emotional understanding, while this effect is not consistently found in girls [101]. High-quality, educational screen content starting around age two may offer developmental benefits, including enhanced cognitive and language skills, improved social attitudes, and enriched imaginative play particularly for disadvantaged children [102]. However, the presence of devices like smartphones in children's personal spaces can interfere with emotional boundaries and lead to overstimulation or emotional detachment. Face-to-face interaction, especially with primary caregivers, remains crucial for promoting social-emotional competence [101]. 

Effective strategies to promote healthy digital media use

Reducing excessive screen time and mitigating digital media usage in children and adolescents require a multi-faceted approach that should be centered on parental awareness, structured guidelines, and collaborative efforts between health and educational authorities [4,18]. Strong evidence shows that parents’ understanding and proactive management of screen habits can significantly lower children’s screen time, which is crucial because early excessive screen use tends to persist and cluster with other unhealthy behaviors like poor diet and insufficient sleep [4,18].

Parental Role and Awareness

Parents are key in shaping screen habits for children and adolescents by setting clear rules and modeling appropriate behavior [103]. Parental restrictions such as limiting access to televisions, computers, and mobile devices, use of parental control settings (e.g., passwords, time limits), and family communication environment are linked to reduced screen exposure in children [104]. Importantly, children living in households where parents spend excessive time watching screens are much more likely to develop similar habits, emphasizing the importance of parental role modelling [105]. In a pilot study in China, Xie et al. (2025) evaluated a multicomponent intervention aimed at reducing children's screen time by enhancing caregivers’ parenting practices and creating a supportive community environment. The intervention significantly reduced total and entertainment screen time on weekends and weekly [106]. In the Growing Up in New Zealand cohort study, it was found that family screen use rules at 24 months were not directly linked to reduced obesity at 54 months but were associated with healthier behaviors, such as lower screen time and longer sleep duration at 45 months. These obesogenic behaviors significantly mediated the relationship between family screen use and child BMI, suggesting family screen use may indirectly help prevent childhood obesity by promoting healthier routines [107]. 

The following principles should be developed to support families in managing screen time and digital media use among children and adolescents, which include:

Screen time restriction: Parents should establish and enforce clear rules on screen duration and timing, with no screen use without permission. For preschool-aged children, combined daily screen time at home and in early childcare settings should not exceed one hour [108,109].

Content monitoring: Media content should be age-appropriate, educational, culturally sensitive, and promote active learning. Caregivers are encouraged to co-view and discuss content with children rather than allowing isolated viewing [24]. 

Developmentally appropriate use: Technology should support a child’s developmental stage and interests without replacing interactive play and physical activity. Active parental engagement during screen use encourages interactive learning and reduces passive consumption, supporting social and cognitive development.

Family media plans: Families should develop customized media plans reflecting household dynamics, including screen-free zones and times such as during meals and before bedtime, to promote balanced daily routines [24].

Inter-Agency Collaborations

Collaboration among the Ministry of Health, Ministry of Education, and social welfare agencies can strengthen efforts to promote healthy screen habits. Parenting programs can be expanded to include education on screen time risks and alternatives, while school health teams can integrate media use assessments and interventions into existing health checks. Support groups for parents may promote the sharing of developmentally enriching, screen-free activities at home [18].

## Conclusions

In conclusion, screen time and digital media use have become deeply embedded in the daily activities of children and adolescents, yielding both benefits and risks. While digital platforms can enhance learning, connectivity, and entertainment, mounting evidence indicates that excessive and unregulated screen exposure may adversely impact physical health through increased risk of obesity, sleep disruption, and visual and musculoskeletal disorders; mental health implications associated with depression, anxiety, self-harm, and declining self-esteem; cognitive development; and social-emotional effects among children and adolescents. Due to the lack of consensus on safe exposure thresholds and the nuanced effects based on screen type and media usage context, a balanced approach is essential. Current global guidelines emphasize limited, high-quality, and supervised screen time, especially during developing years of early childhood. Promoting healthy media habits requires coordinated efforts involving parents, healthcare providers, educators, and policymakers to support the physical, cognitive, and psychosocial development of youth.
